# Statin-Associated Autoimmune Myopathy Masquerading As Recurrent Falls in an Older Adult

**DOI:** 10.7759/cureus.45515

**Published:** 2023-09-18

**Authors:** Malarkodi Suppamutharwyam, Tunku Muzafar Shah

**Affiliations:** 1 Geriatrics, Selayang General Hospital, Batu Caves, MYS

**Keywords:** creatine kinase, recurrent falls, statin, muscle weakness, myopathy

## Abstract

Statins are widely prescribed in clinical practice. Statin-induced myopathy is relatively common, benign, and resolves after statin withdrawal. However, statin-associated autoimmune myopathy is an exceptionally rare and devastating complication that can occur any time after statin initiation. It is characterized by persistent muscle weakness and elevated creatine kinase levels that persist after statin withdrawal. Herein, we present a challenging case of a statin-associated autoimmune myopathy that developed after a decade of atorvastatin use that resulted in debilitating weakness. It is important to recognize cases of myopathies wherein statin discontinuation and aggressive immunosuppressive therapy can reduce morbidity and mortality.

## Introduction

Statins are commonly prescribed for the primary and secondary prevention of cardiovascular and cerebrovascular events [1]. Their mechanism of action involves inhibition of the rate-limiting enzyme 3-hydroxy-3-methylglutaryl-coenzyme A reductase (HMG CoA reductase) in the mevalonate pathway [2]. Statins are effective and have an excellent safety profile [1]. Despite this, approximately 5-10% of patients develop non-specific statin-associated muscle symptoms such as myalgia, myositis, rhabdomyolysis, and asymptomatically elevated creatine kinase (CK) levels [3,4]. These events are benign and self-limiting, and patients usually recover within weeks to months after statin withdrawal. However, some statin-exposed patients develop life-threatening forms of myopathy.

We present a case of a patient with a decade-long history of atorvastatin use who presented with progressive symmetrical proximal muscle weakness, recurrent falls, and elevated CK levels.

## Case presentation

A 76-year-old man was admitted to our geriatric unit with a 6-month history of bilateral progressive muscle weakness involving the upper and lower limbs. Two months prior to admission, he experienced progressive dysphagia and choking episodes whilst eating. He had a 10-year history of hypertension, diabetes mellitus, dyslipidemia, and benign prostatic hypertrophy, and was previously a smoker (20 pack-years). His usual medications included cardiprin (100 mg/day), enalapril (10 mg/day), metformin (500 mg twice per day), terazosin (5 mg/day), and atorvastatin (20 mg nocte). There were no recent changes in his medications. He reported difficulties with standing from a sitting position, walking, climbing stairs, and getting in and out of bed. He suffered four falls previously due to the lower limb weakness. Consequently, he became dependent for basic activities of daily living and was largely bedbound. Further questioning revealed an unintentional weight loss of 20 kg over the previous year and significant fatigue. There was no history of trauma or strenuous exercise, nor any prior history of malignancy or autoimmune disease. There was no family history of neuromuscular disorders. He denied consuming traditional or steroid-containing medications.

On admission, the patient appeared weak and frail, with a body weight of 40 kg. He was dehydrated and delirious. The patient was tachypneic with a respiratory rate of 22 breaths per minute, afebrile, heart rate of 90 beats per minute, blood pressure of 140/90 mmHg, and was able to maintain saturation under ambient air. Physical examination revealed a bilateral upper limb proximal and distal muscle power of 2/5 and 3/5, respectively. In the lower limb, the proximal and distal muscle power were 1/5 and 3/5 respectively. Neck flexion was weak with a power of 2/5. Significant muscle atrophy was observed in the bilateral biceps, interosseous muscles, and quadriceps. Muscle tone, reflexes, and sensation were normal. No focal neurological deficits were observed. The rest of the examination was unremarkable. Laboratory results revealed a markedly elevated CK, aspartate transaminase, alanine transaminase, and lactate dehydrogenase levels (Table 1). Other test results, including electrolyte and hormonal panels, were within the reference range (Table 1). Chest and spine radiography at admission revealed no abnormalities. Electrocardiography showed sinus rhythm. The ear, nose, and throat team were consulted for the dysphagia, and subsequent flexible nasopharyngolaryngoscopy revealed a right vocal fold palsy.

**Table 1:** Progress of the patient’s blood-test results throughout admission. ESR, erythrocyte sedimentation rate; ANA, anti-nuclear antibody; RF, rheumatoid factor; ANCA, antineutrophilic cytoplasmic antibody; PSA, prostate-specific antigen; AFP, alpha fetoprotein; CA 19-9, carbohydrate antigen 19-9; CEA, carcinoembryonic antigen; Hep B, hepatitis B; Hep C, hepatitis C; HBA1c, glycated hemoglobin; HIV, human immunodeficiency virus; VDRL, venereal disease research laboratory; AFB, acid-fast bacilli; MTB, Mycobacterium tuberculosis

Blood parameters	Reference ranges	Day						
		1	2	5	9	11	13	16
White blood cell count (10³/µL)	4–10	8.2	8.1	8.3	9.0	10.0	10.0	23.0
Hemoglobin (g/dL)	12–15	11.2	11.1	9.9	10.6	9.6	9.8	9.1
Platelets (10⁹/L)	150–410	292	290	302	407	477	489	457
C-reactive protein (mg/dL)	0–0.5	0.39	-	26.00	10.00	-	1.75	3.64
ESR (mm/h)	3–5	95	-	-	-	-	-	-
Urea (mmol/L)	1.7–8.3	3.4	2.9	8.5	7.9	8.2	7.9	6.7
Sodium (mmol/L)	135–145	137	138	136	140	148	147	145
Potassium (mmol/L)	3.3–5.1	3.7	3.5	5.0	4.1	3.5	3.6	3.8
Creatinine (µmol/L)	60–120	39	40	30	32	40	38	30
Calcium (mmol/L)	2.02–2.60	2.40	2.23	2.16	2.23	2.15	2.26	2.30
Phosphate (mmol/L)	0.74–1.52	0.93	0.98	0.90	0.93	0.92	0.98	1.06
Magnesium (mmol/L)	0.66–1.07	0.62	0.70	0.71	0.74	0.75	0.78	0.80
Total bilirubin (µmol/L)	3–18	16	14	17	12	12	11	9
Aspartate transaminase (U/L)	1–37	226	191	158	82	90	83	85
Alanine transaminase (U/L)	1–40	238	214	162	144	141	158	160
Lactate dehydrogenase (U/L)	140–271	1,177	1,018	706	434	479	480	472
Creatine kinase (U/L)	25–200	9,185	7,574	1,426	1,472	1,625	2,344	1,016
Thyroid-stimulating hormone (mIU/L)	0.31–3.83	1.5	-	-	-	-	-	-
Free T4 (pmol/L)	12.10–20.20	15	-	-	-	-	-	-
Vitamin B12 (pg/mL)	197–771	350	-	-	-	-	-	-
Folate (ng/mL)	5.60–45.80	5.8	-	-	-	-	-	-
Iron (µmol/L)	8.7–27	12.2	-	-	-	-	-	-
ANA		-	Neg	-	-	-	-	-
RF		-	Neg	-	-	-	-	-
p-ANCA		-	Neg	-	-	-	-	-
c-ANCA		-	Neg	-	-	-	-	-
Complement 3 (g/L)	0.93–1.88	-	1.18	-	-	-	-	-
Complement 4 (g/L)	0.15–0.48	-	0.33	-	-	-	-	-
8 AM serum cortisol (nmol/l)	>350	-	997	-	-	-	-	-
PSA (ng/mL)	0–4	-	3.49	-	-	-	-	-
AFP (ng/mL)	0–9	-	3	-	-	-	-	-
CA 19-9 (U/mL)	0–35	-	33.7	-	-	-	-	-
CEA (ng/mL)	0–5	-	2.2	-	-	-	-	-
HBA1c	<6.5%	5.2%	-	-	-	-	-	-
Hep B and C, HIV, VDRL		Neg	-	-	-	-	-	-
Blood culture		Neg	-	Neg	-	-	Neg	Neg
Sputum culture		Neg	-	Neg	-	-	Neg	Neg
Urine culture		Neg	-	Neg	-	-	Neg	Neg
Sputum AFB		Neg	Neg	Neg	Neg	Neg	Neg	Neg
Sputum MTB culture		Neg	Neg	Neg	Neg	Neg	Neg	Neg

**Figure 1:**
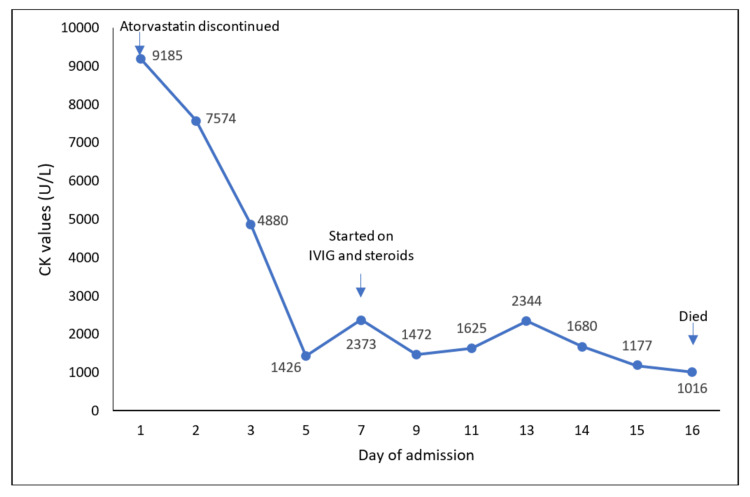
Creatine kinase levels throughout hospital admission. CK, creatine kinase; IVIG, intravenous immunoglobulin.

Upon admission, he was suspected of having either statin-associated rhabdomyolysis or an autoimmune inflammatory myopathy with bulbar involvement. The statin was discontinued immediately, and he was initiated on intravenous crystalloids and nasogastric tube feeding to optimize hydration and nutrition. Enoxaparin (20 mg/day) was initiated subcutaneously for venous thrombo-embolism prophylaxis. His CK levels were closely monitored throughout admission (Figure 1). CK levels improved with hydration. Unfortunately, there was no neurological improvement. Autoimmune rheumatological and myositis panels were then included in investigations. He was scheduled for a nerve conduction study (NCS), electromyography (EMG), and a muscle biopsy.

**Figure 2:**
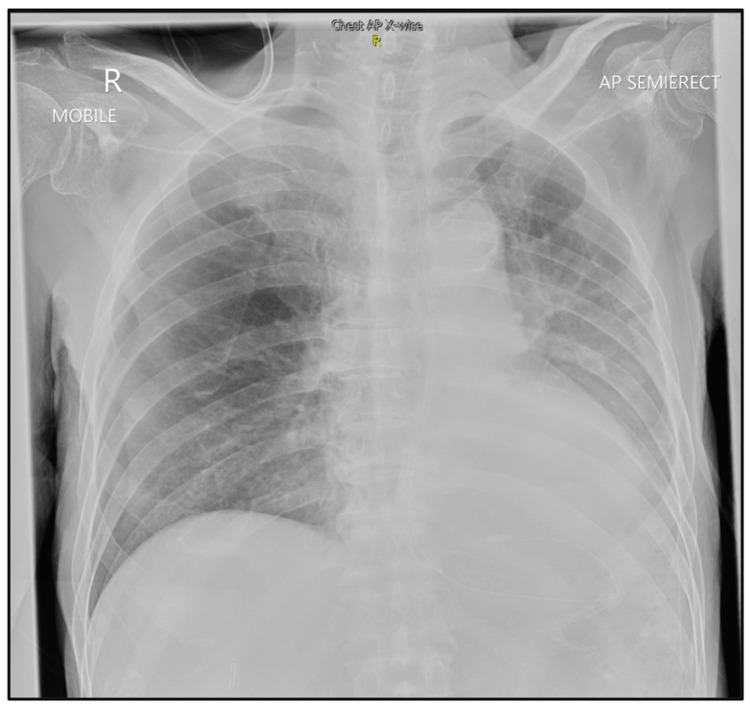
Chest radiograph obtained on day 4 of admission. AP, anteroposterior.

**Figure 3:**
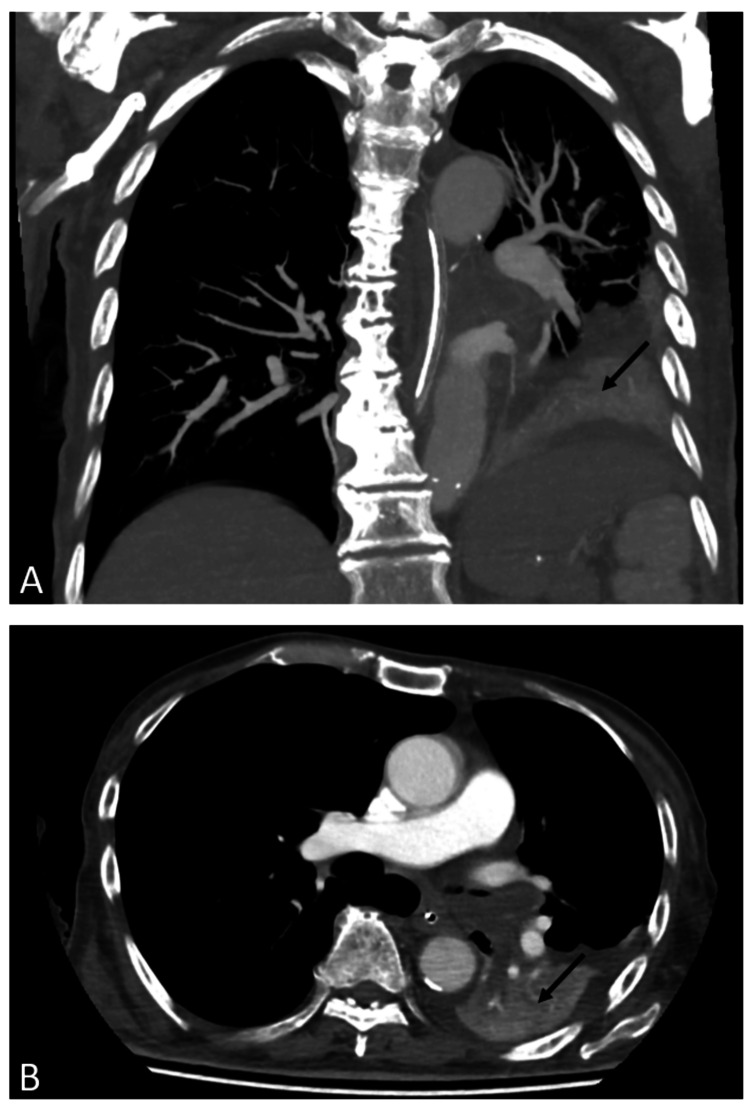
Coronal (A) and axial (B) computed tomography pulmonary angiography show an ill-defined, heterogeneous, hypodense lesion at the anteromedial segment of the left lower lobe (approximate size: 2.6 × 4.3 cm).

Unfortunately, on day four of admission, he deteriorated clinically and developed signs of an infection. He appeared septic, had persistent tachycardia, and required oxygen supplementation. Repeated chest radiography revealed a new consolidation over the right lower zone (Figure 2). He was started on intravenous piperacillin-tazobactam for a nosocomial pneumonia. Computed tomography pulmonary angiography (CTPA) was negative for pulmonary embolism. However, it did reveal an ill-defined, heterogeneous, 2.6 × 4.3 cm hypodense lesion over the anteromedial segment of the left lower lobe, causing the collapse of the left lower lobe (Figure 3). This raised the suspicion of an occult lung malignancy manifesting as an autoimmune myopathy. The respiratory team was consulted for a bronchoscopy.

The patient continued to deteriorate clinically despite receiving high-flow nasal cannula (HFNC) oxygen, antibiotics, and supportive care. Blood, sputum, and urine cultures yielded negative results (Table 1). The patient tested negative for pulmonary tuberculosis, and the autoimmune rheumatological panel was negative (Table 1). All myositis-specific autoimmune serological tests (i.e., for anti-Mi2, MDA5, TIF1, and anti-signal recognition particle) were negative, except for anti-HMG CoA reductase antibodies, which was strongly positive (Table 2). He was diagnosed with a statin-associated autoimmune myopathy. NCS, EMG, muscle biopsy, and bronchoscopy had to be deferred due to clinical instability.

**Table 2:** Results of myositis-related antibody testing. SRP, signal recognition particle; cN-1A, cytosolic 5′ nucleotidase 1A; tRNA, transfer RNA; HMGCR, 3-hydroxy-3-methylglutaryl-coenzyme A reductase

Inflammatory myopathy autoimmune profile	Test	Result
Myositis-specific autoantibodies	Anti-Mi-2 alpha	Negative
	Anti-Mi-2 beta	Negative
	Anti-OJ (Isoleucyl-tRNAsynthetase)	Negative
	Anti-EJ (Glycyl-tRNA synthetase)	Negative
	Anti-PL 12 (Alanyl-tRNA synthetase)	Negative
	Anti-PL 7 (Threonyl-tRNA synthetase)	Negative
	Anti-SAE 1 (SUMO-1)	Negative
	Anti-NXP2/MJ	Negative
	Anti-MDA5/CADM140	Negative
	Anti-TIF 1 gamma (Anti-p155/140)	Negative
	Anti-SRP	Negative
	Anti-Jo 1	Negative
Myositis-associated autoantibodies	Anti-PM-Scl 75	Negative
	Anti-PM-Scl 100	Negative
	Anti-Ku	Negative
	Anti-cN-1A	Negative
	Anti-HMGCR	Strongly positive
	Anti-Ro-52	Negative

Table 3 summarizes the timeline of his presentation. The patient’s presenting symptoms, statin exposure, and thorough physical examination were fundamental in raising the clinical suspicion of a statin-associated autoimmune myopathy or other myopathies such as steroid-induced, polymyositis, dermatomyositis, or endocrine myopathy. Our patient was not on any steroids at presentation. Thus, steroid-induced myopathy was deemed unlikely. Normal thyroid-stimulating hormone levels and the lack of relevant symptoms helped rule out a thyroid-related myopathy. The patient did not have any rashes suggestive of dermatomyositis.

**Table 3:** Timeline of clinical presentation and management.

Six months prior to admission	Bilateral, progressive, and symmetrical proximal muscle weakness in the upper and lower limbs.
Four months prior to admission	Recurrent falls due to bilateral lower limb weakness.
Two months prior to admission	Became dependent for basic daily activities and could no longer move. Experienced dysphagia and choking episodes while eating.
Upon presentation to the emergency department	Assessment and management: Bilateral upper limb proximal and distal muscle power of 2/5 and 3/5, respectively. Bilateral lower limb proximal and distal muscle power of 1/5 and 3/5, respectively. Flexible nasopharyngolaryngoscope revealed a right vocal fold palsy. Laboratory investigations revealed a creatine kinase level of 9185 U/L. Statin was discontinued immediately. Intravenous crystalloids and nasogastric tube feeding initiated.
Day 4 of admission	Developed a nosocomial pneumonia requiring high-flow nasal cannula therapy. Intravenous antibiotics initiated. Autoimmune rheumatological panel was negative.
Day 6 of admission	Computed tomography pulmonary angiography revealed an ill-defined heterogenous hypodense lesion over the left lower lobe, raising suspicion of an occult lung malignancy.
Day 7 of admission	Tests strongly positive for anti-3-hydroxy-3-methyl glutaryl-coenzyme A reductase antibody. Diagnosis of statin-associated autoimmune myopathy with bulbar involvement established. Intravenous immunoglobulin and steroid therapy initiated.
Day 16 of admission	Clinical deterioration due to sepsis and death.

The patient was given intravenous immunoglobulin (IVIG; 0.4 g/kg daily for 5 days) and hydrocortisone (100 mg; three times per day). He tolerated IVIG administration, and the CK level improved. However, no improvement was observed in the proximal or bulbar muscles. Hence, pulsed therapy with intravenous methylprednisolone was planned once the sepsis was under control. Unfortunately, he continued to deteriorate and died on day 16 of admission.

## Discussion

Statin-associated autoimmune myopathy was first described by Christopher-Stine et al. in 2010 [5]. It is rare, with an estimated prevalence of two to three new cases for every 100,000 statin-exposed individuals [6]. Their clinical presentation may comprise acute (within days to weeks) or subacute (within six months) proximal muscle weakness with elevated CK levels. It presents as a progressive symmetrical muscle weakness, leading to atrophy and elevated CK levels despite statin withdrawal [7]. Disease activity is mirrored by the muscle strength and CK levels 10 times above the upper limit normal (ULN), which reflects the degree of muscle cytolysis [8]. In our patient, the CK level at admission was 9185 U/L (i.e., 45 times the ULN). A systematic review by Nazir et al. revealed that 83.33% and 92.8% of the patients presented with symmetrical and bilateral proximal muscle weakness, respectively [9]. Extra-muscular symptoms such as rashes, weight loss, dysphagia, dysarthria, and dyspnea, have also been reported [10]. A systematic review by Somagutta et al. on statin-associated autoimmune myopathy revealed that the mean age of the affected patients was 66±9.4 years and that 61% of the patients were men [10].

The exposure period to statins before clinical presentation of an autoimmune myopathy varied from two months to 10 years, with an average of three years [7]. Evidence supporting the association between the onset of statin-associated autoimmune myopathy and particular statins or dosage effects is limited. Atorvastatin and simvastatin were shown to have a higher propensity for inducing self-limited myopathy [11]. However, an association between specific statins and statin-associated autoimmune myopathy has not been established. In a 25-patient case series on statin-associated autoimmune myopathy, 21 patients were seen exposed to atorvastatin [7]. However, this difference may have resulted from prescribing practices and does not necessarily indicate an association between atorvastatin and autoimmune myopathy. The incidence and severity of statin-induced self-limited myopathy has been postulated to be dose-dependent but this has not been firmly established [12,13].

The exact pathophysiology of this condition is unknown. In 2011, Mammen et al. demonstrated that statin exposure increases the muscle expression of HMG CoA reductase, which presumably triggers an autoimmune response [14]. This autoimmune trigger is sustained by regenerating muscle cells overexpressing HMG CoA reductase, leading to a vicious cycle of muscle damage despite statin withdrawal. Since most patients exposed to statins do not develop autoimmune myopathies, genetic predisposition is thought to play a role. Further research by Mammen et al. in 2012 identified the human leucocyte antigen (HLA) class II allele DRB1*11:01 as a strong genetic factor predisposing White and African American individuals to a statin-associated autoimmune myopathy [15]. Furthermore, HLA class II alleles DQA1 and DQB6 were found to be protective against this condition [15].

The gold standard for diagnosing statin-associated autoimmune myopathy is the detection of serum antibodies against HMG CoA reductase (sensitivity: 94.4%, specificity: 99.3%) [10,16]). Alternatively, if the HMG CoA reductase antibody level cannot be assessed, a diagnosis can be made through a muscle biopsy; these reveal a necrotic rather than an inflammatory state [9]. Magnetic resonance imaging (MRI) of the thighs reveals features of muscle edema consistent with those observed for an inflammatory myopathy. EMG can be utilized to reveal abnormal spontaneous muscle activity with denervation in affected muscle groups, suggestive of an inflammatory myopathy. Our patient was diagnosed with a statin-associated autoimmune myopathy based on symptom history, symmetrical proximal muscle weakness, persistently elevated CK levels despite statin withdrawal, and the presence of serum HMG CoA reductase autoantibodies. Unfortunately, we were unable to proceed with an MRI of the thighs, EMG, and a muscle biopsy due to his clinical deterioration.

No guidelines have been established for the management of this rare disease entity [17]. However, evidence supports immediate statin withdrawal and aggressive immunosuppressive therapy, including steroids [9]. As there are no published randomized controlled trials, the selection of immunosuppressants depends on the patient’s clinical condition, severity of illness, and physician preference [16]. Steroids are considered the first-line therapy, along with steroid-sparing agents such as methotrexate, azathioprine, mycophenolate, IVIG, and rituximab [16]. The aforementioned systematic review by Nazir et al. revealed that 83% of the patients required two or more immunosuppressants during the disease course [9]. Furthermore, the systematic review by Somagutta et al. revealed that 92% of patients returned to their baseline function or experienced symptomatic improvement following treatment with a combination of immunosuppressants [10]; however, 3.75% of the patients died despite treatment [10]. Statin-associated autoimmune myopathy appears to be a chronic disease requiring long-term immunosuppressive therapy along with intensive rehabilitation to restore physical function. Somagutta et al. reported that the average time to remission was 8 months and the time to reach low-dose steroid therapy was approximately 4 months after initial diagnosis [10].

Physicians should be vigilant when subjecting patients to long-term steroid treatment because myopathy can worsen with the concomitant use of high-dose steroids. Steroid-induced myopathy should be suspected if patients demonstrate worsening muscle weakness with ongoing steroid therapy despite improvements in CK levels [18]. Tiniakou reported that the association between statin-associated autoimmune myopathy and malignancy was rather conflicting and ambiguous depending on the geographic location of the patients [16]. The associations were not significant in patient cohorts from Australia, Canada, and the United States, but were significant in cohorts from Japan and France [16]. Therefore, in addition to aggressive immunosuppressive therapy, patients of a certain geographic origin could benefit from age-appropriate malignancy screening. Moreover, the adverse effects of statins should be clearly documented in the patient’s clinical notes to avoid a re-prescription in the future.

Statin-associated autoimmune myopathy is a rare disease entity that has been described in the last 10 years. Further in-depth research is needed to address the following knowledge gaps to improve patient outcomes: 1) whether plasmapheresis is beneficial in patients with severe refractory disease; 2) whether patients can be safely prescribed statins or other lipid-lowering agents (such as ezetimibe or fenofibrates) for secondary prevention; and 3) whether statins can be safely initiated in first-degree relatives or offspring of these patients since genetic factors have been implicated in statin-associated autoimmune myopathy.

## Conclusions

Persistent proximal muscle weakness and elevated CK levels after statin withdrawal should alert one to the possibility of a statin-associated autoimmune myopathy. A comprehensive history (including the timeline of weakness onset and statin exposure), detailed physical examination, and serum HMG CoA reductase antibody levels are required to confirm the diagnosis. Early diagnosis, prompt withdrawal of statins, and immunosuppressive therapy can minimize morbidity and mortality. We advocate the creation of a registry for data synthesis and more research on treatment modalities to improve outcomes.

## References

[1] Feingold KR (2000). Cholesterol lowering drugs. [updated 2021 mar 30]. Endotext [Internet]..

[2] Willey JZ, Elkind MS (2010). 3-Hydroxy-3-methylglutaryl-coenzyme A reductase inhibitors in the treatment of central nervous system diseases. Arch Neurol.

[3] Essers D, Schäublin M, Kullak-Ublick GA, Weiler S (2019). Statin-associated immune-mediated necrotizing myopathy: a retrospective analysis of individual case safety reports from VigiBase. Eur J Clin Pharmacol.

[4] Sathasivam S, Lecky B (2008). Statin induced myopathy. BMJ.

[5] Christopher-Stine L, Casciola-Rosen LA, Hong G, Chung T, Corse AM, Mammen AL (2010). A novel autoantibody recognizing 200-kd and 100-kd proteins is associated with an immune-mediated necrotizing myopathy. Arthritis Rheum.

[6] Shuster S, Awad S (2020). A rare case of statin-induced necrotizing autoimmune myopathy. AACE Clin Case Rep.

[7] Grable-Esposito P, Katzberg HD, Greenberg SA, Srinivasan J, Katz J, Amato AA (2010). Immune-mediated necrotizing myopathy associated with statins. Muscle Nerve.

[8] Bergua C, Chiavelli H, Simon JP, Boyer O, Jouen F, Stenzel W, Martinet J (2016). Immune-mediated necrotizing myopathy. Z Rheumatol.

[9] Nazir S, Lohani S, Tachamo N, Poudel D, Donato A (2017). Statin-associated autoimmune myopathy: a systematic review of 100 cases. J Clin Rheumatol.

[10] Somagutta MK, Shama N, Pormento MK (2022). Statin-induced necrotizing autoimmune myopathy: a systematic review. Reumatologia.

[11] Nichols L, Pfeifer K, Mammen AL, Shahnoor N, Konersman CG (2015). An unusual case of statin-induced myopathy: anti-HMGCoA necrotizing autoimmune myopathy. J Gen Intern Med.

[12] Mohassel P, Mammen AL (2013). Statin-associated autoimmune myopathy and anti-HMGCR autoantibodies. Muscle Nerve.

[13] Thompson PD, Clarkson PM, Rosenson RS (2006). An assessment of statin safety by muscle experts. Am J Cardiol.

[14] Mammen AL, Chung T, Christopher-Stine L, Rosen P, Rosen A, Doering KR, Casciola-Rosen LA (2011). Autoantibodies against 3-hydroxy-3-methylglutaryl-coenzyme A reductase in patients with statin-associated autoimmune myopathy. Arthritis Rheum.

[15] Mammen AL, Gaudet D, Brisson D, Christopher-Stine L, Lloyd TE, Leffell MS, Zachary AA (2012). Increased frequency of DRB1*11:01 in anti-hydroxymethylglutaryl-coenzyme A reductase-associated autoimmune myopathy. Arthritis Care Res (Hoboken).

[16] Tiniakou E (2020). Statin-associated autoimmune myopathy: current perspectives. Ther Clin Risk Manag.

[17] Jasim M, Sapkota H, Timmons M, Manfredonia F, Pohl U, Barkham N (2020). Statin-induced autoimmune necrotizing myositis-a single-center case series highlighting this potentially life-threatening but treatable condition. Clin Case Rep.

[18] Joudeh AI, Albuni MK, Hassen SS, Iqbal P, Aziz Bedair EM, Mahdi S (2022). A case report of statin-induced immune-mediated necrotizing myopathy treatment challenges. Case Rep Rheumatol.

